# Editorial: The Effects of Physical Activity and Exercise on Immune Responses to Infection

**DOI:** 10.3389/fimmu.2022.842568

**Published:** 2022-02-17

**Authors:** Patricia M. L. Dutra, Silvia A. G. Da-Silva, José Roberto Mineo, James E. Turner

**Affiliations:** ^1^ Discipline of Parasitology, Department of Microbiology, Immunology and Parasitology, State University of Rio de Janeiro, Rio de Janeiro, Brazil; ^2^ Laboratory of Imunoparasitology "Dr. Mário Endsfeldz Camargo", Department of Immunology, Institute of Biomedical Sciences, Federal University of Uberlândia, Uberlândia, Brazil; ^3^ Department for Health, University of Bath, Bath, United Kingdom

**Keywords:** physical activity, exercise, immune response, infections, exercise immunology

Protection against infectious disease is provided by innate and adaptive immunity and the magnitude and effectiveness of immune defenses is strongly influenced by a range of host-factors. Since the 1980s and 1990s, there has been a greater appreciation that physical activity and exercise can have a powerful effect on immune function and, in this field named “exercise immunology”, around 5,000 experimental and review articles have been published to date. The influence that physical activity, exercise, and lifestyle, can have on many aspects of immune function has been thoroughly investigated, but some cells, processes, and infections, have received more attention than others. Thus, a concerted effort needs to be made so that understudied topics are examined to understand more about how exercise and other aspects of lifestyle can affect immune function and therefore overall health.

Infectious diseases are among the biggest causes of death in the world. Infections of the lower respiratory tract are the most lethal communicable diseases, causing around 3 million deaths annually. In addition, each year around 1.4 million people die from diarrheal diseases, 1.3 million people die from tuberculosis, 1 million people die from HIV/AIDS, and 435,000 people die from malaria. In 2020, a new infectious disease, COVID-19, whose etiologic agent is the SARS-CoV-2 virus, has, at the date of publishing this article (January 2022), caused more than 5.60 million deaths, having infected around 347 million people (data from 01/25/2022 - https://www.worldometers.info/coronavirus/).

It is well established that structured exercise and habitual physical activity – which might be undertaken as part of normal living – are beneficial for overall health and mental well-being, reducing the risk of chronic disease. Furthermore, elements of an active lifestyle can modulate the immune response to infections and other physiological processes, such as inflammation in metabolic disorders like obesity, diabetes, and cardiovascular diseases. Parameters including the intensity and duration of exercise can differentially influence the immune system. For example, moderate-intensity exercise can stimulate a Th1-type cellular immune response, helping to eliminate intracellular infections, such as viral infections of the upper respiratory tract. Other evidence shows that high-intensity exercise can stimulate a Th2-type response, consisting of an anti-inflammatory cytokine pattern, which might be particularly beneficial for responding to extracellular parasites in addition to the well-known benefits for countering chronic disease. Indeed, the influence that exercise can have on the ability of the immune system to respond to different infections, both intra- and extra-cellular, requires further investigation.

In the case of the protozoa infections with *Leishmania major* and *Trypanosoma cruzi*, regular exercise reduces the severity of diseases in hosts. In the study by Pedra-Rezende et al. (Physical Exercise Promotes a Reduction in Cardiac Fibrosis in the Chronic Indeterminate Form of Experimental Chagas Disease), an exercise program on a treadmill for 4 weeks promoted a reduction in cardiac fibrosis. Although no decrease in parasite load or inflammatory parameters were observed with exercise, the data show that exercise can improve quality of life and prevent the progression of Chagas Disease.

A study by Guimarães et al. (Chronic aerobic training at different volumes in the modulation of macrophage function and in vivo infection of Balb/c mice by *Leishmania major*) examined the effects of light volume (LV - swimming for 30 min, not carrying additional weight, 2 times per week over 10 weeks) and medium volume (MV- swimming for 30 min, carrying a weight of 3.5% body mass, 3 times per week over 10 weeks) exercise. Both forms of exercise impaired a measurement of macrophage infection, referred to as infection factor of macrophage (IF = percentage of infected macrophages × the average number of amastigotes per macrophage) by *L. major*. However, very high volume exercise (VHV - swimming for 60/90 min, carrying a weight of 3.5% body mass, 5 times per week over 10 weeks) showed an increase in IF. The cytokine concentration pattern measured in the supernatants of these macrophages suggested a predominant Th1 response profile in the LV and MV groups. However, Th2 profile predominated in the VHV group and when decreasing exercise volume (tapering [TAP] among mice from the VHV group, so they were swimming for 60 min, not carrying a weight, 2 times per week, over 2 weeks) groups. BALB/c mice infected with *L. major*, that undertook to high-volume (swimming for 60/90 min, carrying a weight of 3.5% body mass, 3 times per week over 12 weeks) exercise, exhibited a significant increase in lesions severity caused by the parasites, in addition to showing visceralization of the infection. The data suggest an increase in susceptibility to parasite infection with high-volume exercise training.

Exercise might be a strategy for improving resistance to infectious disease among at-risk groups such as patients with cancer, as shown by Bartlett et al. (The Effects of 16 Weeks of Exercise Training on Neutrophil Functions in Breast Cancer Survivors). Breast cancer therapy induces prolonged immunosuppression, leaving patients vulnerable to infections. Bartlett et al. showed that a 16-week exercise training program restored, at least in part, the functionality of neutrophils, and, in particular, their phagocytic capacity. Whether these findings translate into a reduction in the risk of infections remains to be established, but this study is an important contribution to understanding the impact of exercise training on immune function in vulnerable patient groups.

Previous research has shown that long-term exercise training can improve responses to vaccines, but it is not well established whether these effects are observed among older adults exhibiting signs of immunosenescence. Felismino et al. (Better response to influenza virus vaccination in physically trained older adults is associated with reductions of cytomegalovirus-specific immunoglobulins as well as improvements in the inflammatory and CD8+ T-cell profiles) demonstrated that older adults who were chronically infected with cytomegalovirus (CMV), exhibit improved vaccine responses when engaging in regular exercise. The study showed that with regular practice of combined exercise modes for 12 months, the IgA specific response to influenza virus vaccination was improved, which occurred in parallel with a higher serum IL-10/IL-6 ratio, and a fall in the ratio of effector to naïve CD8+ T cells.

Previous research has also shown that if an influenza vaccine is administered into the upper arm shortly (i.e., minutes) after acute muscle-damaging eccentric upper arm exercise, antibody responses are improved. However, few studies have confirmed these findings among older adults or made robust measurements of cell-mediated immunity. Elzayat et al. (No effect of acute resistance exercise on immune responses to influenza vaccination in older adults: a randomized controlled trial) show that eccentric upper arm exercise had no effect on antibody response or specific T-cell responses to influenza vaccination, concluding that more strenuous exercise might be required to elicit an adjuvant effect among older adults.

We hope that the readers of this Research Topic will find the articles interesting and useful in this emerging field. This Research Topic emphasizes that exercise can influence the immune system’s ability to mount responses to immune challenge (e.g. vaccination or infection). Here, we see that regular physical exercise helps in the prognosis of parasitic infections, either by helping to reduce cardiac fibrosis promoted in Chagas disease, or by improving the responses of macrophages to *Leishmania* parasite. It may also help restore, at least in part, the functionality of neutrophils and, in particular, their phagocytic capacity in breast cancer patients. On the other hand, in excess, exercise can lead to an increase in experimental infection by microorganisms such as *L. major* In vaccination, exercise promotes better responses to the flu vaccine. However, eccentric arm exercise appears not to affect the antibody or specific T-cell responses to vaccination against influenza virus among older adults. Thus, the magnitude and direction of the effect are determined by the frequency, intensity, and duration of exercise, the period during which the exercise is performed, and the type of immune challenge. This Research Topic includes studies that contribute to advancing the understanding of the mechanisms involved in this complex process and expands our view of how an active lifestyle impacts health.

## Author Contributions

All authors contributed to the article and approved the submitted version.

## Conflict of Interest

The authors declare that the research was conducted in the absence of any commercial or financial relationships that could be construed as a potential conflict of interest.

## Publisher’s Note

All claims expressed in this article are solely those of the authors and do not necessarily represent those of their affiliated organizations, or those of the publisher, the editors and the reviewers. Any product that may be evaluated in this article, or claim that may be made by its manufacturer, is not guaranteed or endorsed by the publisher.

